# A case of twins affected by psoriasis, psoriatic arthritis and autism: Five years of efficacious and safe treatment with Secukinumab

**DOI:** 10.1111/dth.15533

**Published:** 2022-05-04

**Authors:** Nicoletta Bernardini, Nevena Skroza, Federica Marraffa, Elisabeth Prevete, Alessandra Mambrin, Ilaria Proietti, Ersilia Tolino, Martina Caviglia, Antonio Di Guardo, Giovanni Rossi, Salvatore Volpe, Giuseppe Bersani, Concetta Potenza

**Affiliations:** ^1^ Dermatology Unit “D. Innocenzi”, Polo Pontino Sapienza University of Rome Rome Italy; ^2^ Department of Human Neuroscience Sapienza University of Rome Rome Italy; ^3^ Department of Medico‐Surgical Sciences and Biotechnologies Sapienza University of Rome Latina Italy

Autism spectrum disorders (ASDs) are neurodevelopmental conditions having a multifactorial pathogenesis. Thus, beside environmental factors, several twin studies have suggested a polygenic mode of transmission for these disorders.^1^ Autism‐related symptoms include deficit in social communication, restricted and repetitive behaviors and interests, with an impairment in life functioning.^2^ ASDs estimated prevalence is around 1% with a typical onset in childhood, persisting in adult life.^3^ Several psychiatric and medical comorbidities have been reported in ASDs. Psoriasis has been associated as well. In fact, the role of the immune system in ASDs is a research topic of increasing interest, with evidence suggesting that alterations of the immune response and related inflammation mediators^4^ may be involved, but strong evidence is still lacking.

Herein, we report the case of two monozygotic twins, which were diagnosed as ASDs and psoriasis at the age of 4 years. At the time of diagnosis, the first of the homozygote twins (“twin A"—80 kg, BMI 35), had a severe psoriasis affecting the entire body surface, with a Psoriasis Area Severity Index score (PASI) 35; the second twin (“twin B”—78 kg, BMI 34) had a less severe form (PASI 17), with a delayed onset (2 months later). Their medical history revealed that they had both previously been treated with topical therapy (corticosteroid and calcipotriol) followed by methotrexate 15 mg/week, for approximal 1 year, experiencing only a partial response. When assessed at our unit, they were 31‐year‐old men, with a severe psoriasis (respectively PASI 25 and PASI 20) and symptoms of symmetric peripheral polyarthritis, rheumatoid factor negative. Before starting biological therapy, routinary laboratory tests, physical examination, an X‐ray and a neuropsychiatric counseling were performed. The treatment with Secukinumab was started at November, 2016: initially the patients received five subcutaneous (300 mg weekly) injections, followed by once‐a‐month administration. After 4 weeks of treatment, they showed a rapid improvement with a reduction of the PASI score to 9 and 4, respectively for twin A and twin B. At the sixth month, they achieved PASI 0 and a joint pain relief. To date, 5 years after, both twins continue therapy with clinical stabilization, without side effects. Interestingly, twins' mother reported a slight upgrading in their level of attention and sociability following therapy with Secukinumab. This observation was also sustained by improvement in DLQI score (from 28 at beginning to 1 on the last visit). A basic neuropsychological evaluation, also including the analysis of both patients' previous neuropsychiatric history, confirmed such anecdotal report as well. To our knowledge, few articles have been published concerning psoriasis among twins.^5^ Psoriasis in monozygotic twins appears to present a similar age of onset, distribution pattern and severity. Some studies have found that children with ASDs have a higher prevalence of autoimmune diseases including psoriasis, than healthy subjects. Furthermore, different studies have shown an increased IL‐17 serum levels in ASDs that correlates significantly with the severity of autism,^6^ although its possible role in the pathogenesis of ASDs has not been established to date. Interestingly, Nadeem et al showed improvement of autism‐like symptoms in mice through reduction of Th17 immune response.^7^ In our case report, Secukinumab,^8^ an IL‐17A inhibitor, had showed rapid and significant efficacy in the treatment of moderate‐to‐severe psoriasis and psoriatic arthritis (PsA), in twins affected also by autism. Moreover, clinical tolerability and safety were successfully maintained for 5 years, as well as improvement in their psychopathological profile.^9^ To our knowledge, this is the first report of homozygous twins affected simultaneously by psoriasis, PsA and autism, efficiently treated with an anti‐IL‐17A. Further studies are needed to determine the pathogenetic role of IL‐17A in ASDs and the usefulness of an anti‐ IL‐17A therapy in case of this comorbidity.

## CONFLICT OF INTEREST

The authors declare no conflict of interest.

## AUTHOR CONTRIBUTIONS

**Nicoletta Bernardini, Nevena Skroza, Federica Marraffa:** Conceptualization, methodology. **Elisabeth Prevete, Alessandra Mambrin, Ilaria Proietti, Ersilia Tolino:** Data curation, writing—original draft preparation. **Martina Caviglia, Antonio Di Guardo, Giovanni Rossi, Salvatore Volpe:** Visualization, investigation. **Concetta Potenza:** Supervision. **Giuseppe Bersani:** Writing—reviewing and editing.

## Data Availability

Data sharing not applicable to this article as no datasets were generated or analysed during the current study.
